# Supplementary material for: Multi-Steps Fragmentation-Ion Trap Mass Spectrometry Coupled to Liquid Chromatography Diode Array System for Investigation of Olaparib Related Substances
Source: Molecules. 2019 Feb 27;24(5):843. doi: 10.3390/molecules24050843 (PMC6429096; doi:10.3390/molecules24050843)
Supplement: Supplementary file 1 [file molecules-24-00843-s001.pdf]

# Supplementary information

## Multi-steps fragmentation-ion trap mass spectrometry coupled to liquid chromatography diode array system for investigation of olaparib related substances

**Alaa Khedr[1]. Soad S. Abd El-Hay[2]. Ahmed K. Kammouna[1]**

[1] Department of Pharmaceutical Chemistry, Faculty of Pharmacy, King Abdulaziz University, Jeddah 21589, P.O. Box 80260, Saudi Arabia
[2] Department of Analytical Chemistry, Faculty of Pharmacy, Zagazig University, Egypt

IT-MS spectra of OLA and OLA-related substances, $+MS^{2,3}$ and $-MS^{2,3}$

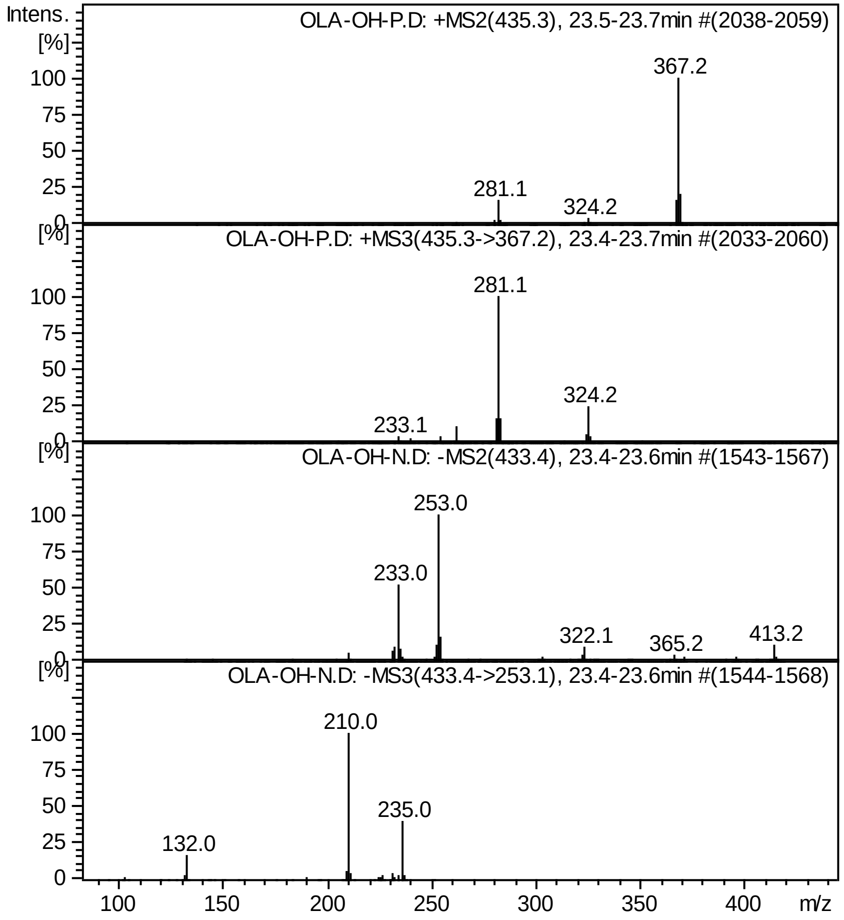


Figure S1: Average negative and positive IT-MS$^{2, 3}$ spectra of *m/z* +435, +435→+367 and -433, -433→253

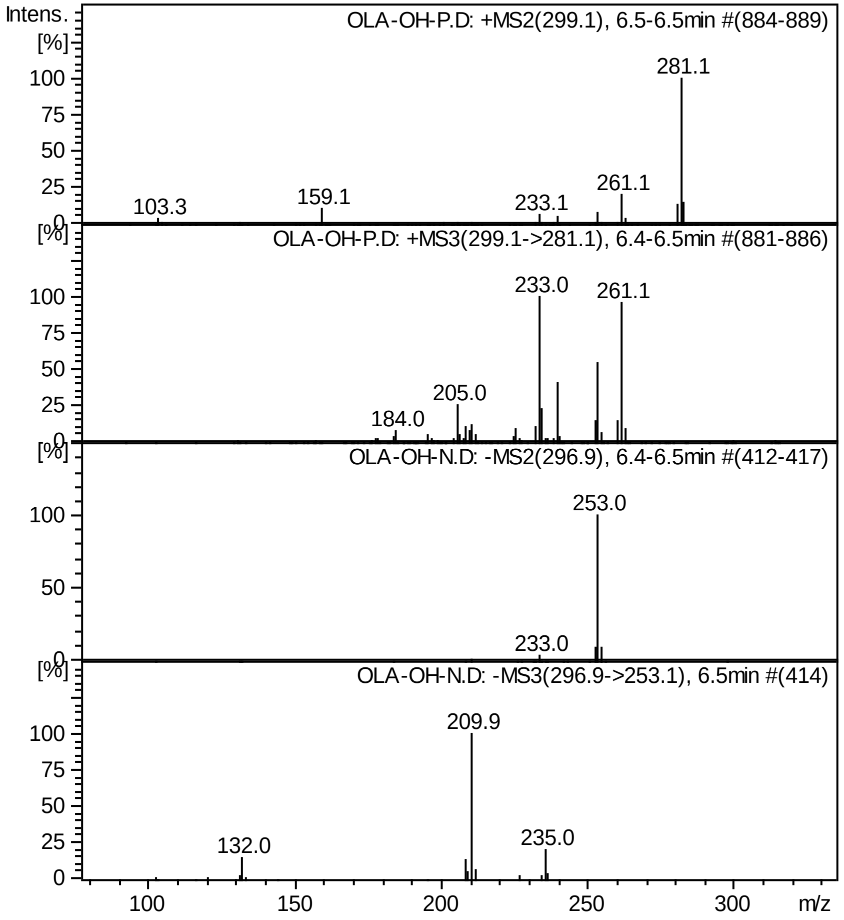


Figure S2: Average negative and positive IT-MS$^{2, 3}$ spectra of *m/z* +299, +299→+281 and -297, -297→253

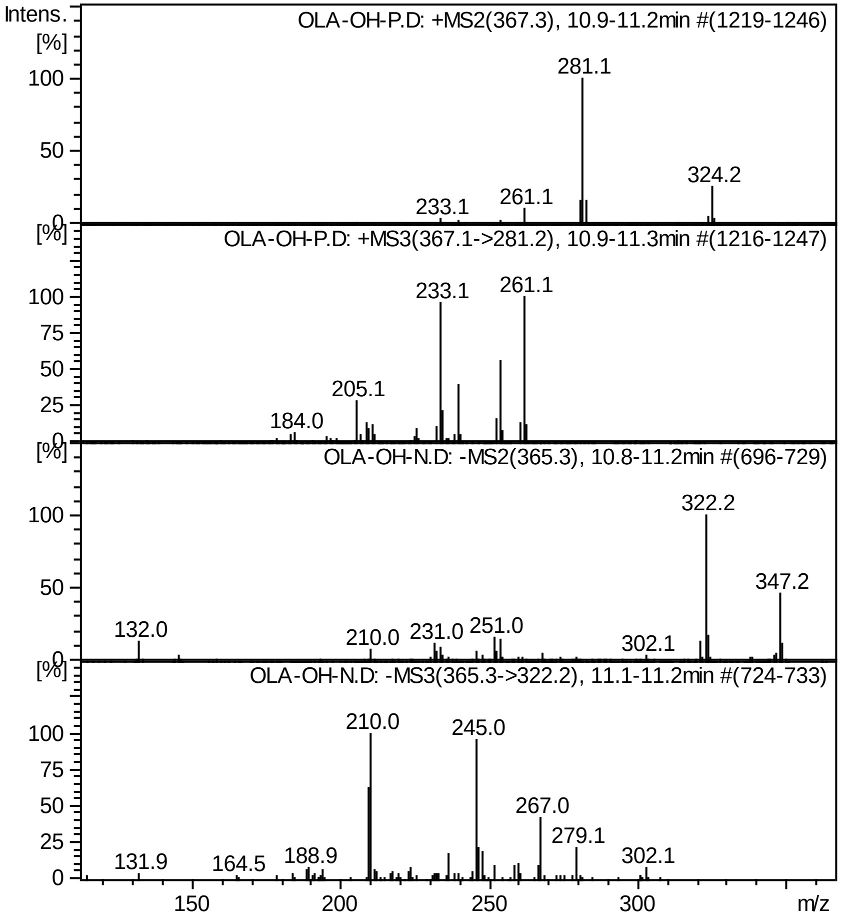


Figure S3: Average negative and positive IT-MS$^{2,3}$ spectra of *m/z* +367, +367→+281 and -365, -365→322.

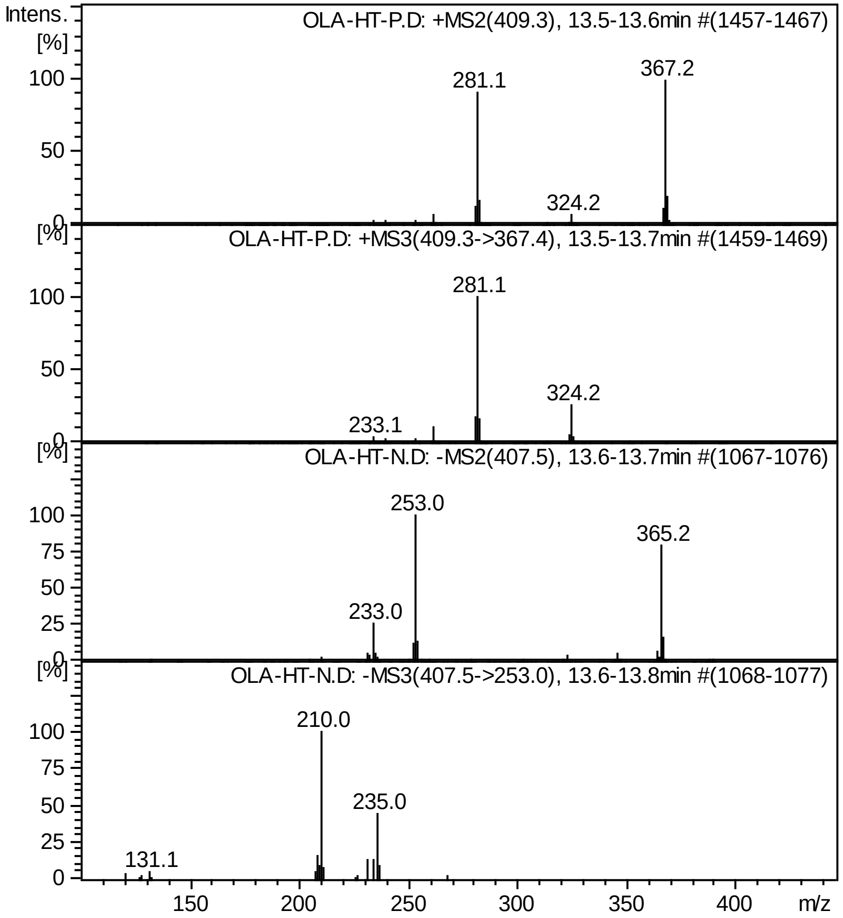


Figure S4: Average negative and positive IT-MS$^{2,3}$ spectra of *m/z* +409, +409→+367 and -407, -407→253.

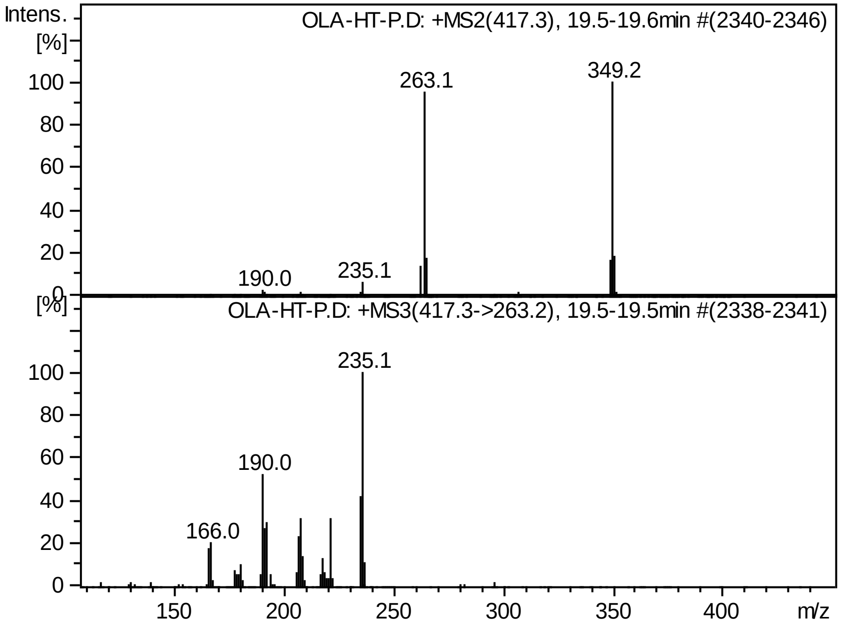


Figure S5: Average positive IT-MS$^{2, 3}$ spectra of *m/z* +417, +417→+263.

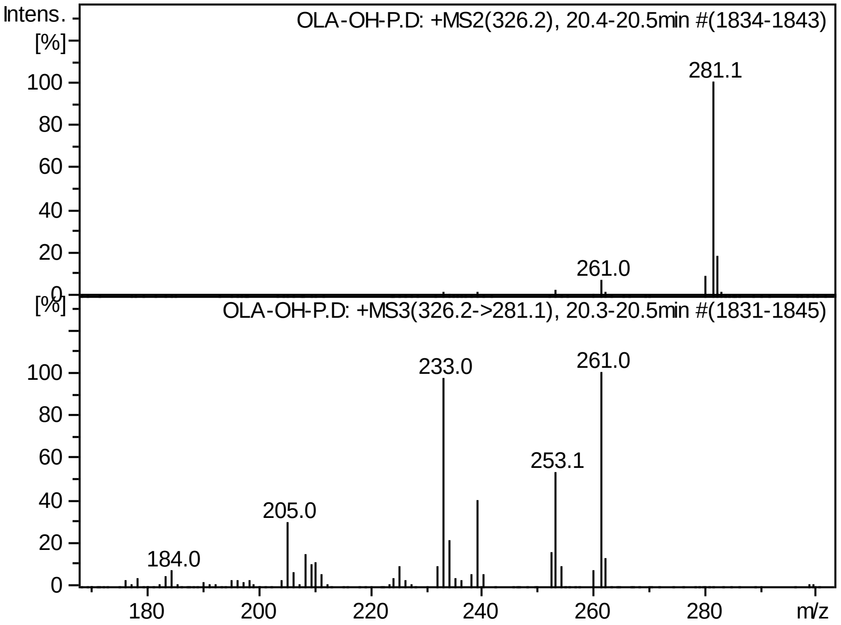


Figure S6: Average positive IT-MS$^{2, 3}$ spectra of *m/z* +326, +326→+281.
